# PyNumDiff: A Python package for numerical differentiation of noisy time-series data

**DOI:** 10.21105/joss.04078

**Published:** 2022-03-22

**Authors:** Floris Van Breugel, Yuying Liu, Bingni W. Brunton, J. Nathan Kutz

**Affiliations:** 1 Department of Mechanical Engineering, University of Nevada at Reno; 2 Department of Applied Mathematics, University of Washington; 3 Department of Biology, University of Washington

## Statement of need

The numerical computation of derivatives is ubiquitous in every scientific discipline and engineering application because derivatives express fundamental relationships among many quantities of interest. As a result, a large number of diverse algorithms have been developed to differentiate numerical data. These efforts are challenging because, in reality, practitioners often have sparse and noisy measurements and data, which undermine the ability to estimate accurate derivatives. Among the diversity of mathematical approaches that have been formulated, many are ad hoc in nature and require significant bespoke tuning of multiple parameters to produce reasonable results. Thus, at a practical level, it is often unclear which method should be used, how to choose parameters, and how to compare results from different methods.

Regardless of application domain, scientists of various levels of mathematical expertise would benefit from a unified toolbox for differentiation techniques and parameter tuning. To address these needs, we built the open-source package PyNumDiff, with two primary goals in mind: (1) to develop a unified source for a diversity of differentiation methods using a common API, and (2) to provide an objective approach for choosing optimal parameters with a single universal hyperparameter (gamma) that functions similarly for all differentiation methods (Van Breugel et al., 2020). By filling these needs, PyNumdiff facilitates easy computations of derivatives on diverse time-series data sets.

## State of the field

Currently, practitioners in need of numerical differentiation tools must often implement a number of methods themselves, before selecting one that is appropriate for their application. High-quality data can leverage computationally efficient and algorithmically simple methods such as the finite-difference, as implemented by standard packages such as NumPy (Harris et al., 2020), SciPy (Jones et al., 2001; Virtanen et al., 2020), or specialized packages like findiff (Baer & others, 2018). Data that are sparse and noisy, however, require more sophisticated algorithms that pracitioners must build themselves based on routines implemented across modules found in disparate packages such as SciPy, PyKalman (Duckworth & others, 2012), PyDMD (Demo et al., 2018), or stand alone scripts such as these implementations of total variation regularization (Chartrand, 2011; Rudin et al., 1992). At present, there is no centralized repository that offers a diverse range of vetted numerical differentiation tools under a unified API in Python, or other software languages.

## Summary

PyNumDiff is a Python package that implements methods for computing numerical derivatives of noisy data. In this package, we implement four commonly used families of differentiation methods whose mathematical formulations have different underlying assumptions, including both global and local methods (Ahnert & Abel, 2007). The first family of methods usually start by applying a smoothing filter to the data, followed by a finite difference calculation (Butterworth, 1930). The second family relies on building a local model of the data through linear regression, and then analytically calculating the derivative based on the model (Belytschko et al., 1996; Savitzky & Golay, 1964; Schafer, 2011). The third family we consider is the Kalman filter (Aravkin et al., 2017; Crassidis & Junkins, 2004; Henderson, 2001; Kalman, 1960), with unknown noise and process characteristics. The last family is an optimization approach based on total variation regularization (TVR) method (Chartrand, 2011; Rudin et al., 1992). For more technical details, refer to Van Breugel et al. (2020). Individual methods under each family are accessed through the API as pynumdiff.family.method.

Applying PyNumDiff usually takes three steps: (i) pick a differentiation method, (ii) obtain optimized parameters, and (iii) apply the differentiation. Step (ii) can be skipped if one wants to manually assign the parameters, which is recommended when computation time is limited and the timeseries is long. Alternatively for long timeseries, optimal parameters can be chosen using a short but representative subset of the data. This optimization routine is provided as a sub-module (pynumdiff.optimize) with the same structure of differentiation families (i.e. pynumdiff.optimize.family.method). By default, the package performs the optimization using the open source CVXOPT package. Faster solutions can be achieved by using proprietary solvers such as MOSEK.

The software package includes tutorials in the form of Jupyter notebooks. These tutorials demonstrate the usage of the aforementioned features. For more detailed information, there is a more comprehensive Sphinx documentation associated with the repository.

## References

[R1] Ahnert K, & Abel M (2007). Numerical differentiation of experimental data: Local versus global methods. Computer Physics Communications, 177(10), 764–774. 10.1016/j.cpc.2007.03.009

[R2] Aravkin A, Burke JV, Ljung L, Lozano A, & Pillonetto G (2017). Generalized Kalman smoothing: Modeling and algorithms. Automatica, 86, 63–86. 10.1016/j.automatica.2017.08.011

[R3] Baer M, & others. (2018). Findiff, a python package for finite difference numerical derivatives and partial differential equations in any number of dimensions. https://github.com/maroba/findiff

[R4] Belytschko T, Krongauz Y, Organ D, Fleming M, & Krysl P (1996). Meshless methods: An overview and recent developments. Computer Methods in Applied Mechanics and Engineering, 139(1–4), 3–47.

[R5] Butterworth S (1930). On the theory of filter amplifiers. Wireless Engineer, 7(6), 536–541.

[R6] Chartrand R (2011). Numerical differentiation of noisy, nonsmooth data. International Scholarly Research Notices, 2011. 10.5402/2011/164564

[R7] Crassidis JL, & Junkins JL (2004). Optimal estimation of dynamic systems (Vol. 2). Chapman & Hall/CRC Boca Raton, FL.

[R8] Demo N, Tezzele M, & Rozza G (2018). PyDMD: Python Dynamic Mode Decomposition. The Journal of Open Source Softw*are*, 3(22), 530. 10.21105/joss.00530

[R9] Duckworth D, & others. (2012). Pykalman, the dead-simple Kalman filter, Kalman smoother, and EM library for Python. https://github.com/pykalman/pykalman

[R10] Harris CR, Millman KJ, Walt S. J. van der, Gommers R, Virtanen P, Cournapeau D, Wieser E, Taylor J, Berg S, Smith NJ, Kern R, Picus M, Hoyer S, Kerkwijk M. H. van, Brett M, Haldane A, Río J. F. del, Wiebe M, Peterson P, … Oliphant TE (2020). Array programming with NumPy. Nature, 585(7825), 357–362. 10.1038/s41586-020-2649-2 32939066 PMC7759461

[R11] Henderson Geoff. T. (2001). Fundamentals of Kalman filtering: A practical approach. The Aeronautical Journal, 105(1049), 400–400. 10.1017/S000192400001232X

[R12] Jones E, Oliphant T, & Peterson P (2001). SciPy: Open source scientific tools for Python. http://www.scipy.org/

[R13] Kalman RE (1960). A new approach to linear filtering and prediction problems. 10.1109/9780470544334.ch9

[R14] Rudin LI, Osher S, & Fatemi E (1992). Nonlinear total variation based noise removal algorithms. Physica D: Nonlinear Phenomena, 60(1–4), 259–268. 10.1016/0167-2789(92)90242-f

[R15] Savitzky A, & Golay MJE (1964). Smoothing and differentiation of data by simplified least squares procedures. Analytical Chemistry, 36(8), 1627–1639. 10.1021/ac60214a047

[R16] Schafer RW (2011). What is a Savitzky-Golay filter? [Lecture notes]. IEEE Signal Processing Magazine, 28(4), 111–117. 10.1109/msp.2011.941097

[R17] Van Breugel F, Kutz JN, & Brunton BW (2020). Numerical differentiation of noisy data: A unifying multi-objective optimization framework. IEEE Access, 8, 196865–196877. 10.1109/access.2020.3034077 33623728 PMC7899139

[R18] Virtanen P, Gommers R, Oliphant TE, Haberland M, Reddy T, Cournapeau D, Burovski E, Peterson P, Weckesser W, Bright J, Walt S. J. van der, Brett M, Wilson J, Millman KJ, Mayorov, Nelson ARJ, Jones, Kern R, Larson E, … Vázquez-Baeza Y (2020). SciPy 1.0: Fundamental algorithms for scientific computing in Python. Nature Methods, 17(3), 261–272. 10.1038/s41592-019-0686-2 32015543 PMC7056644

